# Improving Clinician‐Patient Communication in Aboriginal Health Care: Barriers and Enablers to Implementing Culturally Informed Communication Training (Clinical Yarning)

**DOI:** 10.1111/ajr.70236

**Published:** 2026-09-30

**Authors:** Ivan Lin, Deborah G. Balmer, Charmaine Green Smith, Di Franklin, Kelsie Crowe, Peter O'Sullivan, Jonathan Bullen, Dawn Bessarab

**Affiliations:** ^1^ Western Australian Centre for Rural Health University of Western Australia Geraldton Western Australia Australia; ^2^ WA Country Health Geraldton Western Australia Australia; ^3^ Curtin School of Allied Health, Faculty of Health Sciences Curtin University Perth Western Australia Australia; ^4^ Office of the Deputy Vice‐Chancellor, Indigenous Curtin University Perth Western Australia Australia; ^5^ Centre for Aboriginal Medical and Dental Health, School of Medicine University of Western Australia Perth Western Australia Australia

## Abstract

**Objective:**

This study examines barriers and enablers to implementing Clinical Yarning into health care from the perspective of health care staff.

**Setting:**

The Midwest Region (> 60,000 km^2^), Western Australia (WA).

**Design:**

Semi‐structured interviews with health care practitioners across four regional, rural and remote centers. Interview schedule was drawn from the Theoretical Domains Framework (TDF). Interviews were transcribed verbatim and analysed using inductive and framework analysis, informed by the TDF and Mechanisms of Action.

**Results:**

Interviews with 53 health care practitioners (17% Aboriginal, 83% non‐Aboriginal) ranged from 14 to 60 min in length. Barriers and enablers coalesced around three main themes: knowledge/perceptions of Clinical Yarning, training practices in general and Clinical Yarning specifically, and environmental conditions including general cultural safety and executive support. The barriers and enablers found in each of the domains lead to implementation strategies which use program champions and executive support, build on the positivity towards Clinical Yarning, pay attention to training delivery modes like including local cultural experts where able and acknowledge the larger cultural safety/security barriers within the health service. The barriers presented by workforce challenges are important key variables in any success of the Clinical Yarning intervention.

**Conclusion:**

The documented enablers and barriers to implementing Clinical Yarning in the Midwest when viewed through a TDF/MoA framework provide a way to create a systematic implementation strategy to address the identified barriers and enablers. The findings have implications for other health services wishing to implement Clinical Yarning as well as culturally secure health care more broadly.

## Key Points

**What is already known on this topic:**

- Implementing a skills‐based communication training into mainstream healthcare that acknowledges the specific cultural needs of Indigenous Australians is challenging.
- Many of those barriers and enablers have been generally documented in the literature and others may be unique to the implementation context and training focus.

**What this study adds:**

- Clinical Yarning implementation was influenced by three key factors: staff knowledge and perceptions of Clinical Yarning, training approaches—generally and Clinical Yarning specifically and organizational/environmental conditions.
- The use of a Theoretical Domains Framework/Mechanisms of Action approach identified practical strategies to address barriers and strengthen enablers.
- This study may provide a template for others to use the same framework to plan and execute an implementation project for communication training.

## 1 Background

Effective communication between patients and health care practitioners (HCPs) is the foundation for safe, high quality health care that is valued by patients [1, 2, 3]. Improving health care communication is a priority consistently reported by Aboriginal and Torres Strait Islander people (henceforth respectfully referred to as Aboriginal reflecting the Western Australian location of the study) across diverse health settings and health conditions including cancer services [4] chronic diseases [5], pain [6] and emergency care [7]. For many Aboriginal patients, ineffective communication with HCPs results in patients not being appropriately informed about their health condition(s), unmotivated to engage in care, mistrusting of health services and disengaged from care [6, 8, 9]. Effective communication, alongside a well‐resourced Indigenous health workforce, trusting relationships [10] and supportive health care systems that value Indigenous peoples' cultural knowledge, beliefs and values, is critical to culturally safe health care [3]. Improving clinical communication in Aboriginal health care is therefore an important priority.

Clinical Yarning is a framework that has been developed as a patient‐centred communication approach in Aboriginal health care [11]. Clinical Yarning (CY) was adapted from the seminal work of Bessarab and Ng'andu in research yarning [12]. Clinical Yarning re‐frames clinical communication as a social, diagnostic and management yarn, mirroring the reciprocal, two‐way, conversational informal communication approach used by Aboriginal people when yarning [8]. Further details of CY and its background are explored fully in earlier work [8]; however, in summary, the CY program focuses on equipping health care providers with practical communication skills to enhance their clinical practice. CY preferences patient connectedness and building trust. Training to support HCPs in a CY approach is offered through in‐person workshops in small group settings with or without online CY eLearning modules. Previous studies evaluating clinicians' perspectives reported strong support for CY and increased clinician self‐reported knowledge, confidence and skills communicating with Aboriginal patients [13, 14].

The Midwest region of WA covers an area of 605 000 km^2^ with a population of over 54 500 people. The region is a quarter of WA's land mass, with most of the population resident in one larger regional city. The Midwest area in the study is the land of the Yamatji peoples; Wajarri is the predominant language. Health services in the region include regional, district and small hospitals, health centres and remote area clinics [15]. Approximately 10% of the population in the Midwest Region are Aboriginal and/or Torres Strait Islander [16]. Improving the health of Aboriginal people is a priority for the local health service [17, 18].

The paper reports on the first phase of a project to implement CY in the Midwest region, Western Australia (WA). The study takes a systematic implementation science approach to explore barriers and enablers to CY. This information was used to plan an implementation strategy and we anticipate findings from that will be useful to other health services interested in implementing CY or other programmes to improve communication or cultural safety.

## 2 Method

The overall CY project is a four‐phased implementation [19] study (see Figure 1). This paper reports on one aspect of the first phase, a qualitative investigation of the barriers and enablers to implementing CY across the Midwest region [see [15] for details] using semi‐structured interviews with health care staff, either individually or as part of a small group.

**FIGURE 1:**
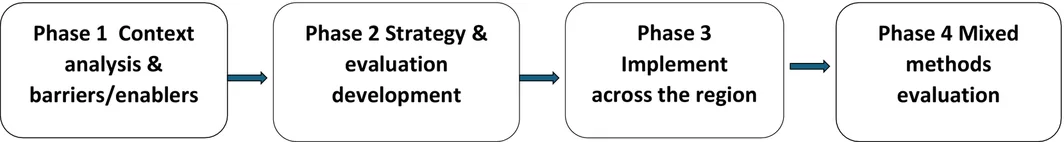
Overall Clinical Yarning Implementation Project.

Health staff from across the government health service in the Midwest were recruited using purposive sampling [20]. Initially, an organizational map was developed in order to reflect different regions, areas within the health care service and professional groups. Participants were sought from nursing, medical, allied health, Aboriginal Liaison Officers and managerial areas with the invitation, participant information sheets and consent sent via email. Some participants were also followed up by phone. Signed participant consents were returned prior to interviews or consented at the interviews. A focus was also made to include, where possible, self‐identifying Aboriginal staff working in any of these areas within the Midwest. The aim was also to recruit from across six sites within the Midwest area (classified using the Australian Government's Modified Monash Model (MMM) 2023 [21]) and to get a broad range of views from as broad a range of staff as possible, with stratification used to ensure differing levels of seniority and professional group inclusion.

Ethics approvals for this study were gained from WA Health Central Human Research Ethics Committee (#RGS5764), the WA Aboriginal Health Ethics Committee (#1217) and the University of Western Australia Ethics Committee (#ET000489). The research is funded by a grant from the Future Health Research and Innovation Fund, Implementation Science Fellowship (ISF2021), Department of Health, Western Australia and was a collaboration with the Western Australian Country Health Service (WACHS).

### 2.1 Research Team and Cultural Governance

A central principle of this work was the ‘built‐in’ processes to the way research was conducted to ensure the cultural rights, values and expectations were upheld and promoted [17]. The project team included Aboriginal researchers (CGS, DB, JB, TP, KMC) as well as other researchers with a range of experience in Aboriginal health research (senior to developing) (IL, PO, DGB, DF, KC). Team members directly involved in field work (TP, IL, DGB, KC: 2 male, 2 female) brought expertise and experience in implementation science, qualitative research and interviewing, physiotherapy, education/teaching, health literacy and Aboriginal cultural knowledge. Two advisory groups were integral to guiding the work throughout: an Aboriginal Community Advisory Group (CAG) consisting of six Aboriginal community members and a Stakeholder Advisory Group consisting of health service and academic partners which included Aboriginal members. The local Aboriginal community‐controlled health service offered strong support for the project including a staff member on the Stakeholder Advisory Group.

### 2.2 Data Collection and Analysis

Initial intentional contact to build an organizational schemata took place with many of these initial contacts known to the lead investigator owing to years of residence in the study area. Recruitment therefore of participants and associated logistics was done by DGB, a relative newcomer to the district with few prior connections. An interview schedule (Data S1) was developed collaboratively, using the 14 domains in the Theoretical Domains Framework (TDF) to structure the exploration into barriers and enablers from clinician and health staff perspectives [22, 23]. The TDF is a synthesis of 33 theories of behaviour and behaviour change, used for examining implementation of new practices in healthcare [18]. The TDF enables a comprehensive way to explore the determinants of clinician behaviour and provides a theoretical lens through which to understand health care practices, develop implementation approaches, evaluate change and replicate elsewhere (See Table 5 for domains).

Inclusion criteria for participant interviews were (a) employee of the government health service, (b) over the age of 18, (c) from a range of health professionals in the region, reflected in the organizational map developed initially, and a role either patient‐facing or in health service management. Participants were excluded if they did not meet these criteria. Interviews were conducted in participants' workplaces, either in person or virtually. Participants were interviewed individually or alongside and with colleagues, reflecting internal working relationships. Field notes were kept after some interviews to both record events and detail researcher reflexivity. The research consent form gave participants the option to review their own transcript, which no one accepted. The interview schedule was reviewed together with the research team prior to research commencing. Often there were two researchers at the interviews, which was part of capacity building as well as supporting consistent interpretation across the group. Researcher reflection and de‐briefing sessions were held throughout the data collection phase to reflect on the interviews and to support consistency of interview practice. The interview schedule itself (Data S1) was not adjusted during the data collection, but adjustments were made to interview technique, for example, pausing or using the prompts better for more specific responses. Interviews continued until both our purposive sample aims were reached and further data collection was yielding few additional insights.

Data analysis began concurrently with interviews, allowing the back and forth movement between the existing data and reflection on ways to improve on data quality as collection progressed [20]. No repeat interviews were conducted. Participants had the option of agreeing to receive a summary of the study. Interviews were audio recorded and transcribed using a paid transcription software [24] followed by detailed checking and revision by the researchers, before being de‐identified.

Our objective was to identify barriers and enablers to implementing CY and link these to their underlying behavioural determinants, to be used later to identify suitable implementation interventions or ‘behaviour change techniques’. Analysis started initially with inductive methods where transcripts of interviews were examined for potential dominant coding categories prior to applying the pre‐defined framework used in the deductive methods. Deductive methods [25] were applied using the Theoretical Domains Framework [26] and 12 additional theoretical constructs identified that influence behaviour [22] giving, in total, 26 Mechanisms of Action (MoA), for example, values, general attitudes/beliefs. MoAs are defined as the actions or processes an implementation strategy takes to achieve the desired outcomes [27]. Transcripts were mapped on to these to explore the behaviours which may be barriers or enablers to implementing Clinical Yarning. Initially three interviews were selected as coding moderation samples [28, 29] and coded separately by researchers with interpretations discussed and coding agreed on where there was variation. Researchers thus became thoroughly familiar with the data set, having collected the data, checked transcription and noted observations throughout this process. Emergent codes were discussed amongst several members of the research team as a way to deepen investigation of the data. Headline data categories were discussed with the Community Advisory Group and stakeholders to elicit responses and feedback. Frequent review continued to resolve uncertainties in the coding against the MoAs and arrive at MoA informed themes (Tables 2, 3, 4).

## 3 Results

Semi‐structured interviews took place between August 2023 and February 2024. Fifty‐three people were interviewed in 34 interviews. Eight interviews were participant groups with sizes ranging from 2 to 8 people. Two participants were invited to participate but failed to respond or refused. The total audio‐recorded interview data was 16 h and 35 min with interviews/sessions ranging from 14 to 60 min (average length 29 min). Audio‐recorded individual and group interviews were analysed together as one data set and not distinguished according to group size. Interviews ranged across the intended multiple professional groups, including nursing, allied health, medical, Aboriginal liaison officers, healthcare assistants, health management/administrators from across and representing the 6 regions of the project (Table 1).

**TABLE 1:** Participant descriptors.

Profession/Area of work	Number	%
Nursing	20	38
Medicine	6	11
Allied health	6	11
Mental health/alcohol and drug	8	15
Professional/patient support	6	11
Executive Management	7	13
Sex		
Female	51	96
Male	2	4
Self‐identified as Aboriginal		
Yes	9	16.9
No	44	83.1
Remoteness		
MMM 3	42	79
MMM 4–7^a^	11	21

^a^ Australian Government Department of Health Disability and Ageing [21]. Percentages may not total 100% due to rounding.

The six geographical areas were represented with two of the more remote areas represented by staff whose areas of responsibility covered these areas. There were 9 participants who self‐identified as Aboriginal. Ethnicity data was not formally collected. The number of participants in the study was dictated by both the project timing deadline and adequate participant coverage across the region and specialties.

The barriers and enablers for the implementation of CY coalesced around three main themes: knowledge/perceptions of Clinical Yarning, training practices in general and CY specifically, environmental conditions including general cultural safety and executive support.

### 3.1 Knowledge/Perception of CY

Generally, there was a neutral to positive response towards the proposed CY training regardless of the specialty area. However, knowledge of the content of CY was limited or vague (Table 2, subtheme a). Any prior knowledge generally came from clinical staff who were in more senior roles and who had worked for longer periods of time in the region. When participants did not have any background knowledge of Clinical Yarning, many associated it with engagement with Aboriginal patients as well as cultural awareness more generally. This need for cultural awareness was seen as especially important in remote/rural areas where fly‐in‐fly‐out or agency staffing was commonplace and knowledge of the local cultural context was absent (subtheme c). Some nurses saw CY as having to learn a rigid script and ‘just another training program’, while maintaining that working effectively with Aboriginal patients was best learnt on the job through experience (subtheme d).

**TABLE 2:** Knowledge and perceptions of CY.

Theme	Sub‐theme	Evidence
Knowledge/perception of Clinical Yarning	Limited/vague but generally positive	*So I'm positively inclined towards it, but it is still a very vague thing to me. (SO38)* *It's really valuable training, and particularly for a lot of our staff do not come from an Indigenous background. (S040)*
	b Associated with Aboriginal patients/cultural awareness	*If an Aboriginal person presented to ED or something, you know, a busy nurse who's not used to talking to Aboriginal people, doesn't know the community. There's so many agency staff here, that they wouldn't necessarily just see the big picture for what's going on. (SO16)*
	c Answer to cultural awareness	*(I) Heard from other staff members, when an Aboriginal person walks in the door, some of the different cultural people are. …they see that person and think oh, …they're already feeling uneasy. …because they haven't dealt with those people in the past very well or very, very much. And so they find it hard. (S09)*
	d Rote learning not effective	*I don't think you can give me a script and say this is Clinical Yarning. Because everyone does it differently. And I think that you need to it's an it's an experience based thing. …I still think most of what you learn is experienced based. (S028)*
	e Teaching culture	*With me, I don't really know my…, you know, my cultural things… cos I've been shored up with white people most of my life—younger days and that. …but if we can get someone who can sit down with me and explain things. Yeah… (S006)*

For one participant, however, CY training was seen as an opportunity to learn their own culture, having grown up and lived away from culture (subtheme e).

### 3.2 Training & CY

Participants' views varied on what the enablers and barriers to implementing CY training for health staff might be. There was a clear preference for face‐to‐face training, as opposed to on‐line training (Table 3, subtheme a). Face‐to‐face delivery of CY also meant local, trusted Aboriginal cultural expertise could be included. This was seen as critically important, especially by those in more senior roles (Table 3, subtheme b) and reflected knowledge of the distinctiveness of different Aboriginal cultural groups in different regions, as well as an enthusiasm for more connection to local Aboriginal knowledge holders. There was however an acknowledgement that on‐line learning options allow flexibility and ease of access (subtheme a). In addition, the possibility of gaining educational credit points (e.g., continuous professional development—CPD) from professional registration bodies was mooted as a potential enabler (e.g., medical specialties/nursing/allied health professional bodies).

**TABLE 3:** Training.

Theme	Sub‐theme	Evidence
Training & Clinical Yarning	Face‐to face delivery vs. on‐line	*Recently, everything's been online …So it's good to now introduce it* [face to face] *back in*. (S05) *Face to face is good, …They get more out of it, more interaction, it's more fun than just sitting at a computer (S010)* *Notwithstanding the advantage of flexibility that online learning gives But if it's online, they can do it when the kids have gone to bed or in the morning (S007)*
	b Involvement of local Aboriginal cultural expertise	*But I would love to see, especially for community staff, or anyone really, but more and more face to face more local knowledge—Local input—About our community. Yeah. And that Aboriginal people that live here and that were going to present to us. (S016)*
	c Staff turnover & high use of agency staff in regional areas	*We tend not to train agency staff. …they're paid a lot of money to come already. So if we wanted them to have this training, then we would expect the agencies to provide the training to them (S019)* *Another barrier for us is train the trainer because of the turnover and… I guess redeploying them to do that is a barrier for us. …they tend to not be sustainable (S20)* *Things are not done so well, from a whole service perspective. And especially up here where there's a lot of high staff turnover, there's a lot of processes that really aren't very good. I've seen a lot of stuff, which is [main town] centric. (S036)*
	d Staff time constraints and cost	*It's not like we're [nursing] an office‐based industry, that you can just turn the computer off and go to training, you know (S19)* *We would not have a budget for facilitators—that would just be coming out of their clinical budget (S20)* *I suppose it comes down to economics sometimes as well. …But how much is it going to cost them? And what's the time costs? (S13)*
	e Training champion	*I think it's just, … getting that person who, you know, really runs with it. To keep that momentum going. You know, he talks about it in team meetings, and he talks about it in clinical reviews that I've used this, and these, you know, and then get that buy in from other people. (S018)*
	f Mandatory training	*What I can tell you is there's a large number of things that are mandatory, and it's a huge list, …many of which remain uncompleted because of time pressure. (S033 Allied health)* *Just maybe the eye rolling and that, Oh, God! And the, Don't we already do this? And the, I don't know—just the verbal horizontal violence, they call it? You know…. the culture of the workplace sometimes… Yeah. Becoming a target… (S04)* *For some people (it should be mandatory), …people who have emigrated to Australia…and, you know, anyone who kind of is going to have, you know, Indigenous, as their cohort group…. Renal have a huge Indigenous population, ED perhaps? (S04)*

A significant barrier to training was workforce shortages, staff turnover and, as a result, the high number of agency nursing staff (staff employed on short term contracts through an external workforce agency) in the region, especially in remote sites (subtheme c). Ideally, agency staff should arrive at the location already prepared, including cultural training. However, the need for better trained agency staff, more attuned to the cultural needs of Aboriginal patients, was highlighted especially by senior nurse participants.

Funding arrangements, however, placed the responsibility of training agency staff with the contracting agency, for which the public health system pays a premium. There was a divide between employee types (permanent versus agency) and what training was mandatory for government employees, coupled with the problem of high staff turnover.

Some participants felt the lack of sophistication for provision of cultural security and was a ‘one size fits all which is statewide’ (S036), especially as it relates to cultural training provision.

Another barrier to implementing CY was limited time for training, especially where participation in face‐to‐face training requires staffing backfill and thus adds additional cost. Because of staff turnover, a train‐the‐trainer approach to the training was not seen as viable, with some citing previous cases where it had failed because of that (subtheme d). However, the notion of a ‘champion’ arose as an enabler for CY—champions already employed within the health service with strong support and commitment to seeing CY implemented (subtheme e).

Participants expressed mixed views on whether mandating (i.e., compulsory) CY training would assist implementation (subtheme f). Overall, most participants supported not mandating CY training because there was a large amount of existing mandatory training which exceeded the allocated time available to complete. Wanting to do the training rather than ‘having to do the training’ was viewed as more attractive by most of the respondents, but not all. One Aboriginal participant felt that making the face‐to‐face training mandatory may provoke a backlash from any colleagues with underlying negative attitudes towards a training specifically aimed at communication with Aboriginal patients. However, for staff who were new and/or working in areas of health care with a high number of Aboriginal patients, some saw CY as essential and thus should be mandatory.

### 3.3 Cultural Safety/Security

A significant and unexpected barrier, especially for remote populations, was seeing support for Aboriginal cultural security not embedded systematically into the health care service (Table 4, subtheme a). Others saw a lack of an overall system for cultural safety as the legacy of health services that were established to serve exclusively the needs of the dominant cultural group. Some anticipated institutional barriers for CY because of the prejudicial views of individual staff members. Though CY training addresses one aspect of culturally safe care, it is the system as a whole which was seen as requiring attention (subtheme a).

**TABLE 4:** Cultural security/safety.

Theme	Sub‐theme	Evidence
Cultural Security/Safety	Non‐Systematic approaches to Aboriginal cultural support, especially in remote populations	*I don't see [government health department] having a whole lot of sophistication here on this. I mean, I look at how the ALO here operates in the lack of I wouldn't say that, I don't think she's well supported. I've raised a number of issues here that she's seen as a transport officer. Um there's the lack of processes underpinning what actually happens… I mean… the following up and having those yarn with the patients and a feedback mechanism that comes in—that doesn't exist… (S036)* *We get a lot of [*Aboriginal*] people who come in who DAMA—who don't wait… there's not really any, any systemic follow up for that for any issue… So if it's gonna be just simply it's all back on the clinician to be… for this to be successful. I have issues with that, because it needs to be a whole of service aspects… I think embedding it… where it is focused on within the within all the services is absolutely essential. Because… there's very little… from a cultural awareness, cultural provisioning perspective that I see actually occurring. (S036)* *Lot of them [Indigenous Liaison Officer role] don't get training though. Let me point that out. A lot of them get put into the role simply because they're Indigenous, and they don't get any training into what's expected. (S028)* *You know that there's ALOs around and they come and see the patient, like, you're doing another job, or they see the patient… And you don't learn anything from that. And like so, I think seeing (the patient) together is actually good. The only time I've seen like, together is in like an MDT (multi‐disciplinary team) when we're trying to get a patient home. But no other time together. (S24)*
	b Legacy health systems	*Then you go to [*Rural/remote town*], and nothing. We've got services in place that are so old. And it only is tailored for the white man. So why would you want to accept a service where you've got someone coming into your home and not understanding? Or saying this is what we offer? Well how about we … that might not be beneficial for them? You know? Yeah, I just think that we* (government health service) *haven't really evolved. (S002)* *I really think there's a role in having an understanding why it's been so difficult to engage with Aboriginal people. And a lot of that's true, is trust. It's institutional racism. And, you know, these, these old buildings are not built, they're not equipped. And there's a lot of history and a lot of trauma. (S002)*
	c Practice in Aboriginal health care	*You don't look at a person and say you're Aboriginal, or you're whatever. You look at them and say let's work as a team… Whereas if someone walks in the door, I don't see an Aboriginal person, I just see a sick person. (S009)* *As soon as they get a sense of, umm, like a, like an Aboriginal patient's upset, they will call us straightaway, you know, instead of trying to, resolve it first, I guess. And then it could be as like, simple as just communicating with them and letting them know what's actually going on. (S012)*
	d Staff capacity	*And just thinking too it would actually, it does make sense for it to be the ALOs to do the training, you know, like on site. (S037)* *We try not to, you know, over‐extend our Aboriginal Health Workers either because they're already out in the community doing so much for us, probably more, working really, really hard. (S039)*
	e Influence & attitude from prior learning/experience	*I have been through so many different types of cultural training… I don't think we need to be sat through and told again, how awful we are… I think we just need the nuts and bolts of how can we do this better… How can we engage Aboriginal people? Because after all, the result in particular, what we're chasing is collaboration, cooperation and an outcome. We want a really good health outcome (S030)*

The lack of systematic backing for the cultural support (Aboriginal Liaison Officer—ALO) role, both in scope and training was noted by some. Institutional cultural insensitivity was also seen to relate to historical trauma and mistrust (subtheme b) and where improving Aboriginal health was not viewed as a priority, as evidenced by some witnessing the on‐going delays in a long‐planned new health care facility servicing an area with a high Aboriginal population. The expectation that ALOs would take on the cultural trainer role in the CY implementation seemed unrealistic given the work load, expectations, limited number of ALO staff and large variations in employment and training histories. In addition, some participants questioned whether the health service sufficiently supported ALO roles within the organization to work most effectively.

Conversations about CY evoked broader reflection on engaging with Aboriginal patients in clinical practice more generally. The importance of working with Aboriginal staff who worked in health care was highlighted by some. Alternatively, a few health professionals did not see a patient's Aboriginality status as something requiring a specific approach in their clinical practice, rather it was to be guided by the clinical condition being presented (subtheme c). Some participants reported negative views on prior cultural training experiences which did not include a practical component (Table 4 Subtheme e).

The role of the ALOs arose repeatedly in the discussion around cultural awareness, cultural support and especially in relation to being involved in training and supporting Clinical Yarning. One participant expressed the desire to work together with ALOs while providing patient care in order to learn. However, that was countered by others cautioning against further burdening an already over‐stretched workforce (subtheme d). While discussing the role ALOs could have in Clinical Yarning, another observer highlighted the large variation in training that ALOs receive.

These various insights into ALO role and function in the clinical space are both a barrier and an enabler. The expectation that ALOs would take on the cultural trainer role in the CY implementation seemed unrealistic given the workload, expectations, limited number of ALO staff and large variations in employment and training histories (subtheme d). In addition, some participants questioned whether the health service sufficiently supported ALO roles within the organization to work most effectively (Subtheme a).

## 4 Discussion

In our study we utilized the TDF to guide interviews with staff and to map emergent themes against domains of the TDF/MoA during analysis (Table 5). Enablers were identified and incorporated into Clinical Yarning training, including involving local Aboriginal cultural leaders in program delivery, offering face to face training (as opposed to online), linking training to CPD/CME points, not mandating training and utilizing an existing learning management system to arrange training workshops. Most of these enablers were within a ‘motivation’ behavioural domain [22]. Partnering with local cultural knowledge‐holders has been recommended to improve cultural safety and Aboriginal health care communication [30, 31]. Other enablers included a positive attitude towards Clinical Yarning, overall support from health service executives and identifying some staff as clinical champions of the program (general attitude/beliefs, social influence and motivation domains).

**TABLE 5:** Summary table of themes against TDF/MoA domains.

Theme	Subtheme(s)	TDF/MoA
Knowledge & perceptions about Clinical Yarning	Barrier: lack of awareness of Clinical Yarning	Knowledge
	Enabler: Clinical Yarning – positive attitude (A number of staff hold a positive regard towards Clinical Yarning)	General attitude/beliefs
Training	Barrier: Previous cultural training – negative experience & unconvinced of need for training	General attitudes/beliefs
	Barrier: Cultural training – lack of system esp. for agency staff	Environment context and resources
	Barrier: Negative experience with simulation learning	General attitudes/beliefs
	Enabler: Professional requirements for ongoing CME – Avenue for CY evaluation is requirement to do reflective practice	Motivation
	Enabler: Face to face program preferred	Motivation
	Enabler: Involve local trusted Aboriginal cultural leaders	Motivation
	Enabler: There is executive support (mostly)	Social influence
	Enabler: There is an existing learning platform to organize CY training	Environmental context and resources
	Barrier: Siloed approach to training across and between divisions	Environmental context and resources
	Enabler: Clinical Yarning training not immediately mandatory for existing staff	Social influence
	Barrier: Limited time allocated to training (nursing staff PD time is already committed to mandatory training)	Environmental context and resources
	Barrier: Sustainability challenges due to staff turnover (nursing/medical), remote areas	Environmental context and resources
Train the trainer	Barrier: Staff turnover/Insufficient staff or interest in train‐the‐trainer	Environmental context and resources Motivation
	Enabler: Some staff ‘champions’ are interested in becoming trainers/facilitators	Motivation
Cultural safety within the health service	Barrier: Insufficient ALOs	Environmental context and resources
	Barrier: Insufficient strategic integration of ALO role within the health system	Environmental context and resources
	Barrier: Perceptions (and experiences) of racism within health service	General attitudes/beliefs
	Barrier: Lack of system to support cultural safety	Environmental context and resources
	Enabler: Some staff are optimistic that cultural safety is improving	Optimism

*Note:* TDF Domains: (1) knowledge; (2) skills; (3) social/professional role and identity; (4) beliefs about capabilities; (5) optimism; (6) beliefs about consequences; (7) reinforcement; (8) intentions; (9) goals; (10) memory, attention and decision processes; (11) environmental context and resources; (12) social influences; (13) emotion; (14) behaviour regulation. Mechanisms of Action: Norms, subjective norms, attitude towards behaviour, motivation, self‐image, needs, values, feedback processes, social learning/imitation, behavioural cueing, general attitudes/beliefs, perceived susceptibility/vulnerability.

Several important barriers were identified within an environmental context and resources domain that were beyond the scope of our project. These included workforce challenges with staff shortages and high turnover, especially in remote areas, and limited time that can be allocated to training due to workforce demands, especially for nursing staff. A high number of agency nursing staff, especially in remote areas, was another barrier as, given the short‐term nature of their work and the extra costs to the health service, agency staff are not supported to undertake training. These findings reflect well established challenges in Australian remote and rural health care [32] and are an additional challenge when aiming to implement new health care approaches in this context.

Other significant barriers within the domain of environmental context and resources and general attitudes/beliefs were related to cultural safety more broadly. This included an insufficient number of Aboriginal staff, limited integration of ALOs within the health system, a lack of system to support culturally safe care more broadly and perceived racism within the health service. Clinical Yarning/communication can build culturally safe care, alongside a well‐resourced Indigenous health workforce, trusting relationships, and supportive health systems that include physically welcoming spaces that address racism [31]. In our project we are targeting communication through Clinical Yarning; however, we do not know if a communication‐focused project, without attention to other elements is sufficient to result in health care change given that these factors are interlinked as part of cultural safety. Nor is it clear whether by addressing communication, there is the potential to influence other aspects of cultural safety.

By identifying barriers and enablers and their associated behavioural domains, we can develop suitable interventions based on evidence‐supported behaviour change techniques to implement in the next project stage [23]. For example, some participants expressed previous negative experiences of cultural training and were unconvinced Clinical Yarning would be different, thus potentially impacting on the uptake of Clinical Yarning training. This barrier was aligned to the behavioural domain of general attitudes/beliefs. One evidence‐supported behaviour change technique to address general attitudes/beliefs is to present alternative information from a credible source [23]. Therefore, our strategy will be to develop a communication strategy with senior staff (credible source) [33] that reinforces a positive attitude towards Clinical Yarning, distinguishes it from cultural training, reinforces the goal of the project and describes the positive outcomes from previous research findings. By mapping barriers and enablers to behavioural domains and then behaviour change techniques, we can develop a systematic implementation plan that we will re‐visit during the evaluation phase to determine what, why and how interventions did or did not work. Theory‐informed approaches such as this are needed in cultural interventions so as to increase understanding of what works when and to maximize change [34].

Several implementation strategies have been previously reported in Aboriginal health care clinical communication research. These include strategies to increase utilization of Aboriginal interpreter services in Australian Northern Territory hospitals [35, 36], and intercultural exchange and co‐creation of educational process and resources between Yolnu (First Nations Australians from North‐East Arnhem Land) and non‐Indigenous staff from education, health or community services [37]. One intervention, a multifaceted approach to enhance communication in three Northern Territory hospitals was underpinned by one implementation theory, the COM‐B [38] to ensure interventions broadly addressed each area of the model (i.e., capability, opportunity and motivation) although interventions were not tailored in response to data collected in the project [30]. Clinical Yarning contrasts to previous work because it adopts a behavioural‐skills approach which explicitly aims to change clinician communication practices [11]. Evidence suggests that Clinical Yarning is highly valued by clinicians [11, 14] and results in more effective communication practices [39]. Ultimately, further implementation‐oriented research to improve Aboriginal health care communication is needed in order to understand what interventions are effective, in what context, so these can be applied successfully elsewhere. Further research is also needed on Aboriginal patient experiences of Clinical Yarning.

This study identified a range of barriers and enablers to implement Clinical Yarning within a regional Western Australian health service. Effective communication is a foundation to culturally safe/secure health care [40] and a priority in Australian health care [41]. Although there has been extensive documentation of communication issues in Aboriginal health care, most studies to date have either described these issues [42, 43] or proposed frameworks/models to improve communication [44]. Fewer studies have implemented strategies to enhance communication, with fewer still taking a theory‐informed approach [44] to this issue. This study applied a theory‐based approach to investigate barriers and enablers to implementing Clinical Yarning which will be used to develop implementation interventions/strategies. Findings are relevant to health services and researchers wishing to enhance clinical communication and cultural safety, and we hope to advance efforts to improve Aboriginal health care clinical communication more broadly.

## 5 Limitations & Strengths

The strengths of this study are the diverse perspectives each member of our team contributes as community members, Aboriginal and other researchers, clinicians and health managers to this topic and our systematic approach to advance an important and under‐researched area. There is a broad cross section of health care staff, from different professional groups and geographic sites who participated. A potential limitation is that some rural/remote sites may have unique place‐based opportunities and challenges that may not have been identified. Another potential limitation is the predominance of females in the sample group, and whether findings would have been different if the male/female participant numbers were more balanced. Likewise, there is also an over‐representation of the nursing profession; however, the spread of positions and responsibilities across the organization reflects a predominance of females in those roles making recruitment of male participants not possible. There is also potential for bias (relational, response‐shaping) as the research was conducted in small communities, where researchers are often embedded in that community and thus known to participants. Separate reporting of Aboriginal perspectives was not appropriate because of the small sample and potential identification risks. Further, the application of the TDF/MoA in Aboriginal health research is relatively new. In time, implementation theoretical frameworks that integrate Indigenous approaches with Western implementation theories may be more suitable in Aboriginal health services research.

## 6 Conclusion

Taking a systemic theory‐informed approach, we identified important enablers and barriers to implementing Clinical Yarning across a Western Australian regional health service. Identified enablers, including involving local cultural experts, supporting program champions and building on positive regard for the topic and barriers such as prior negative experiences of cultural training, will be used to plan what, how and when to deliver evidence‐informed interventions. A number of environmental/contextual barriers were identified, including a lack of overall cultural safety within the health service and workforce challenges that are beyond the immediate scope of the project and could potentially impact on the success of the interventions. The findings and theoretical approach taken are relevant to health services wishing to improve clinical communication (and cultural safety) and to deepen understanding of how to make change in these areas.

## Author Contributions

**Ivan Lin:** conceptualization, investigation, funding acquisition, writing – original draft, methodology, validation, writing – review and editing, formal analysis, supervision, project administration. **Deborah G. Balmer:** investigation, writing – original draft, methodology, writing – review and editing, software, formal analysis, project administration. **Di Franklin:** methodology, writing – review and editing, resources, supervision, funding acquisition, formal analysis. **Peter O'Sullivan:** conceptualization, methodology, writing – review and editing, supervision, funding acquisition. **Charmaine Green Smith:** investigation, conceptualization, funding acquisition, writing – original draft, writing – review and editing, supervision. **Jonathan Bullen:** conceptualization, funding acquisition, methodology, writing – review and editing, supervision. **Kelsie Crowe:** writing – review and editing, resources, project administration. **Dawn Bessarab:** funding acquisition, conceptualization, methodology, writing – review and editing, resources, supervision.

## Funding

This study is a three‐year Implementation Science Fellowship (2021) (ISF2021‐LIN). funded by the Western Australian Future Health Research and Innovation Fund, Department of Health, Government of Western Australia.

## Ethics Statement

Ethics approvals for this study were gained from Western Australia Health Central Human Research Ethics Committee (#RGS5764), Western Australian Aboriginal Health Ethics Committee (#1217) and the University of Western Australia Human Ethics Office (#2023/ET000489).

## Conflicts of Interest

The authors declare no conflicts of interest.

## Supporting information


**Data S1:** Supporting Information.

## Data Availability

The data that support the findings of this study may be available on reasonable request from the corresponding author, subject to relevant ethics approvals, data custodian requirements and Aboriginal governance approvals.
