# Supplementary material for: Improving Clinician‐Patient Communication in Aboriginal Health Care: Barriers and Enablers to Implementing Culturally Informed Communication Training (Clinical Yarning)
Source: Aust J Rural Health. 2026 Sep 30;34(5):e70236. doi: 10.1111/ajr.70236 (PMC13627743; doi:10.1111/ajr.70236)
Supplement: Supplementary file 1 [file AJR-34-0-s001.docx]

**Focus Group/Discussion Guide for clinicians/health leads & ALOs**

This discussion aims to explore the barriers and enablers to the two proposed changes in health care:

1. Clinicians within (****) utilising a Clinical Yarning approach to communication with Aboriginal patients
2. Staff to deliver regular, sustainable Clinical Yarning training and support via a train-the-trainer approach

**Procedure for focus groups/individual interviews**

1) Acknowledgement/Welcome to Country
2) Consent & any clarification of PIS
3) Study overview and goals of the session –

a) to gather views on barriers and enablers to clinicians using the Clinical Yarning approach to communicate with Aboriginal patients

b) to assess the viability of using in-house trainers for Clinical Yarning training/train the trainer approach – and

4) Present vignettes from Aboriginal patients’ experiences from our research

| **Demographic Details** |  |
| --- | --- |
| Position/Job _____________ | Participant # ___________________ |
| Region __________________ | Date ________________________ |
|  |  |

| Question | Prompts | TDF domain(s) |
| --- | --- | --- |
| Have you heard the term clinical yarning previously? | - Associations? - Contexts? | Optimism  Emotion  Knowledge |
| If yes: tell us what you understand about clinical yarning? | - CY Structure/approach - Use of/experience with CY | Knowledge |
| If no: tell us what your thoughts are about communication with Aboriginal patients in the healthcare context | - Knowledge about communication - Previous training? - Skills? - Confidence? associated emotions? important to you/important to colleagues/important to health service? - What help or hinder effective communication – - How do feel about communication in Aboriginal health care? | knowledge  skills  capability  beliefs about consequences/social-professional role/intentions-goals/social influences/  intentions  reinforcement  emotion |
| To roll out Clinical Yarning across (workplace), we are planning to use a train-the-trainer approach. Have you seen this model work for professional development training?  What worked?  What didn’t? | - What knowledge/skills are needed - How confident this is possible - Who’s role is this? - How do you feel about a train-the-trainer approach - What resources are needed? Is this available? - What training and support would be needed? - What management support exists/is needed? - Does the culture within the workplace support this? Why/why not? - Sustainability in the workplace of on-going training with workforce turn-over - Interest in becoming a trainer – who has face validity to be a trainer? - Other delivery models? E.g., mix on-line/face to face delivery | Knowledge/skills  Capabilities  Professional role  Emotion/optimism  Environmental context & resources  Social influences |
| What other strategies could support or hinder clinical yarning/improved communication in (workplace)? | - Additional support for staff? If so, what type? - Leadership/commitment within workplace? - Incentives, reminders? - Organisational support, resourcing, culture, policies? | Knowledge, skills.  Social/professional role/identity  Reinforcement, memory, attention and decision processes  Environmental context and resources |
